# The Cognition of Maximal Reach Distance in Parkinson's Disease

**DOI:** 10.1155/2016/6827085

**Published:** 2016-08-15

**Authors:** Satoru Otsuki, Masanori Nagaoka

**Affiliations:** ^1^Department of Rehabilitation, Juntendo University Nerima Hospital, 3-1-10 Takanodai, Nerima-ku, Tokyo 177-8521, Japan; ^2^Department of Rehabilitation Medicine, Juntendo University Graduate School, 2-1-1 Hongo, Bunkyo-ku, Tokyo 113-8421, Japan

## Abstract

This study aimed to investigate whether the cognition of spatial distance in reaching movements was decreased in patients with Parkinson's disease (PD) and whether this cognition was associated with various symptoms of PD. Estimated and actual maximal reaching distances were measured in three directions in PD patients and healthy elderly volunteers. Differences between estimated and actual measurements were compared within each group. In the PD patients, the associations between “error in cognition” of reaching distance and “clinical findings” were also examined. The results showed that no differences were observed in any values regardless of dominance of hand and severity of symptoms. The differences between the estimated and actual measurements were negatively deviated in the PD patients, indicating that they tended to underestimate reaching distance. “Error in cognition” of reaching distance correlated with the items of posture in the motor section of the Unified Parkinson's Disease Rating Scale. This suggests that, in PD patients, postural deviation and postural instability might affect the cognition of the distance from a target object.

## 1. Introduction

In daily life, we frequently perform movements such as extending a hand toward an object. Such movements are expressed as reaching movements. The reaching movement is composed of postural control and transport of the upper limbs and hands. These elements are considered to be automatic processes. Regarding the association between the reaching movement and postural control, the functional reach test developed by Duncan et al. [1] determines the maximal reaching distance and uses postural stability as an indicator. Before the reaching movement is actually performed, there is also an essential cognitive process in which people visually perceive a target object and decide how to reach it. Presumably, people estimate how far they can spatially reach on the basis of information from the visual, auditory, and somatic senses and their past experiences and then start movement on the basis of this estimation [2, 3]. The distances that study participants estimate to be reachable through this cognitive process can be measured, and there are several reports on this subject [4–10].

This reaching movement is limited by diseases affecting the movement of the upper limbs and postural control. The reaching movement of patients with Parkinson's disease (PD) has also been described in many reports, and the reported characteristics of their reaching movement include the delay of the start of movement [11], slow speed of movement [12], and impaired coordination between the arm and the trunk [13]. Moreover, in PD patients, the reaching distance determined by the functional reach test is shorter in those with a history of falls than in those without [10, 14, 15], and the reaching distance is suggested to be associated with decreased postural stability and falls. It is assumed that, in PD patients, reaching movement is limited by decreased movement of the upper limbs due to akinesia or bradykinesia, decreased flexibility due to rigidity of the four limbs and trunk, and the impairment of postural reflexes associated with these decreases. Moreover, it has been reported that PD patients experience disorders in eye movement [16–18] and coordination between the eyes and head with reaching movements [19–21]. Presumably, decreased motility of the eyes, head, and neck due to PD may affect the ability to detect the accurate position of a target object in space.

Regarding the estimated reaching distance, in studies on PD patients, Kamata et al. [9] reported that overestimation of reaching distance due to progression of symptoms is associated with falls. On the other hand, some reports showed PD patients tend to underestimate spatial distance [10, 22, 23]. Factors affecting the cognition of spatial distance have considered physical factors and environment factors [4–6]. Ehgoetz Martens et al. [24] reported that the cognition of spatial distance differs in experimental conditions in PD patients. But the association with detailed motor symptoms of PD has not yet been clarified. These issues of underestimation or overestimation are related factors that need more investigation and are still open to discuss. The difference in the right and left sides of the spatial cognition has been discussed in PD patients [22, 23, 25, 26]. In PD patients, although estimated reaching distance was analyzed only front reach [9, 10], the differences in the right and left sides need more investigation. Thus, regarding the distance estimated in reaching movement by PD patients, we evaluated distance cognition in a more spatial manner by measuring estimated and actual maximal reaching distance in the right and left directions, in addition to the front direction. Furthermore, the association with the symptoms of PD was also investigated.

While various terms are used to indicate estimated reaching distance, we use the term estimated reaching distance (ED) in the present study. Correspondingly, the term actual reaching distance (AD) is used to indicate the actually reachable distance.

## 2. Subjects and Methods

### 2.1. Subjects

The subjects were 27 PD patients (8 men and 19 women; mean age ± standard deviation (SD): 71.9 ± 5.3 years, mean height ± SD: 155.5 ± 7.6 cm) and a control group of 28 healthy elderly volunteers matched for age and height (10 men and 18 women with a mean age ± SD: 73.6 ± 5.2 years and mean height ± SD: 157.1 ± 8.7 cm). All participants were right-handed. In the case of PD patients, those who provided consent for the objectives and contents of the study were included if they could remain standing for long enough to perform the tasks and had cognitive function sufficient to understand the tasks (24 or higher on the Mini-Mental State Exam [MMSE]). The present study was conducted with the approval of the ethics committee of our institution (Ethics Approval Number 13-29).

### 2.2. Methods

In the PD patients, the following data were collected: disease duration, Hoehn-Yahr scale, the motor section of the Unified Parkinson's Disease Rating Scale (UPDRS3), and history of falls. Moreover, the medications that the PD patients were receiving were identified. All patients were treated with levodopa and measurements were taken under the effects of the medications (on-stage). No patients showed dyskinesia during measurement.

**Figure 1:**
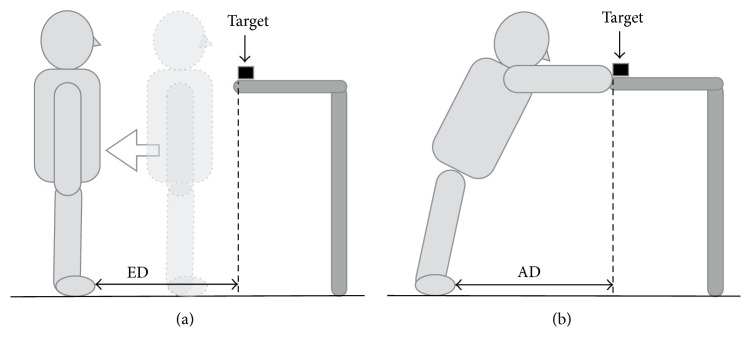
(a) Measurement of front ED. Participants stood on the starting position (dotted line). They stepped backward and stopped at the farthest point where they decided that they could grasp target (solid line). The distance from target to stopping point was regarded as ED. (b) Measurement of front AD. Participants reach out and grasp the target. The distance from target to farthest standing position was regarded as AD.

In the experiments, both ED and AD were measured by the following method. Regarding the AD measurement, functional reach test was verified as reliable [1]. The reliability of ED measurement was verified by Robinovitch and Cronin [7] but was not sufficient. The measurement of ED was performed three times and an Intraclass Correlation Coefficient (ICC) was used for the intrarater reliability. According to the method developed by Fischer [5], ED was measured under the following conditions. The participants estimated the range in which they could grasp a 3 cm square wooden block on a plate at the level of the shoulder in the standing position without taking a step, in the front, right, and left directions. In order to investigate differences in spatial cognition due to directional effects, ED was measured in not only the front direction but also the right and left directions. During measurement, the participants were prohibited from extending their arms or tilting their bodies and measured the distance only by sight. For estimation of the front reaching distance, the participants stood in front of the block placed at the level of their right acromion. This position was regarded as the starting position. They were instructed to slowly step backward in a straight line from any point where they could grasp the block with certainty and to stop at the farthest point where they decided that they could grasp it with both feet touching the floor. The point at the tip of the right big toe was marked, and the distance from the intersection point between the vertical line from the block and the floor to the marked point was measured and regarded as the front ED (Figure 1(a)). For estimation of the right reaching distance, the participants stood in a way that the block placed at the same position as in estimation of the front ED was positioned immediately lateral to the right acromion. This position was regarded as the starting position. They were instructed to slowly step leftward in straight line from any point where they could grasp the block with certainty and to stop at the farthest point where they decided that they could grasp it with both feet touching the floor. The point at the distal end of the right fifth metatarsal bone was marked, and the distance from the intersection point between the vertical line from the block and the floor to the marked point was measured and regarded as the right ED. Estimation of the left reaching distance was performed in the same manner, and the results were regarded as the left ED.

After ED was measured in the three directions, the farthest range in which the participants could actually grasp the block was measured in the three directions in the order of the front, right, and left directions by the following method based on the multidirectional reach test, which Newton [27] had developed by modifying the functional reach test for multidirectional measurement. The participants were instructed to stand at the points marked during measurement of ED and to grasp the block without moving their feet (Figure 1(b)). The block was moved by 1 cm at a time away when they could grasp it or closer when they could not until the farthest point where they could grasp it without stepping out was determined. In this manner, AD was measured in the front direction (front AD), the right direction (right AD), and the left direction (left AD).

### 2.3. Data Analysis

The measurements obtained from the functional reach test have been shown to correlate with body height [1]. Thus, in data analysis, each measurement (i.e., ED and AD) was divided by body height, and values were adjusted for individual differences in body constitution. In order to analyze the differences between estimated and actual measurements, the differences between ED and AD (ED−AD) were calculated. In the present study, these differences are expressed as the difference between ED and AD (DEA). Absolute values of DEA were used for analysis to assess the degree of the differences between ED and AD.

In the control group, the right and left sides were regarded as the dominant and nondominant sides, respectively, to perform data analysis. In the PD patients, the symptomatically milder side (MS) and severer side (SS) were determined according to the total scores obtained from the scores on the items of UPDRS3 for the right and left sides. When the total scores were the same, the affected side at the time of onset was determined as the SS. Each measurement (i.e., right ED, left ED, right AD, and left AD) was classified by MS and SS into MS-ED, SS-ED, MS-AD, and SS-AD.

In order to assess the association between each measurement and its directionality, Pearson correlation coefficients were calculated for each measurement obtained from the dominant and nondominant sides in the control group and the MS and SS in the PD patients (*p* values were corrected by the Bonferroni correction, *p* < 0.012). Moreover, a one-way analysis of variance (ANOVA) was performed with ED, AD, DEA, and absolute values of DEA in each direction (the Bonferroni correction was used for the post hoc test).

In both groups, total values of measurement in the three directions were calculated for ED (total ED) and AD (total AD), and their mean values were compared between the two groups by unpaired *t*-test. In order to evaluate errors in cognition, the mean value was compared between total ED and total AD within each group by paired *t*-test. Initial significance level was set at 0.05 (*p* values were corrected by the Bonferroni correction, *p* < 0.012).

As for PD patients, a stepwise linear regression analysis was performed to identify clinical symptoms associated with errors in cognition. Total DEA obtained from DEA in the three directions and absolute values of total DEA were used as target variables. The following items were used as explanatory variables: MMSE, disease duration, Hoehn-Yahr scale, history of falls, UPDRS3 total score, and individual items of UPDRS3 (tremor: 20, 21; rigidity: 22; diadochokinesis: 23–26; posture: 28, 30; walking: 29; and akinesia: 31). Significance level was set at 0.05.

For all statistical analyses, SPSS version 21 was used.

## 3. Results

### 3.1. Characteristics of PD

Mean and standard deviation (SD) of each item were 27.6 ± 1.8 for MMSE, 5.9 ± 3.3 years for disease duration, 2.6 ± 0.7 for Hoehn-Yahr scale, and 16.0 ± 9.5 for UPDRS3. The number of falling subjects was 12 and the number of nonfalling subjects was 15.

### 3.2. Reliability of ED Measurement

A result of the reliability verification of ED measurement showed ICC (1,3) = 0.953; therefore it is considered as reliable method.

### 3.3. Validity of the Methods and Measurements

**Figure 2:**
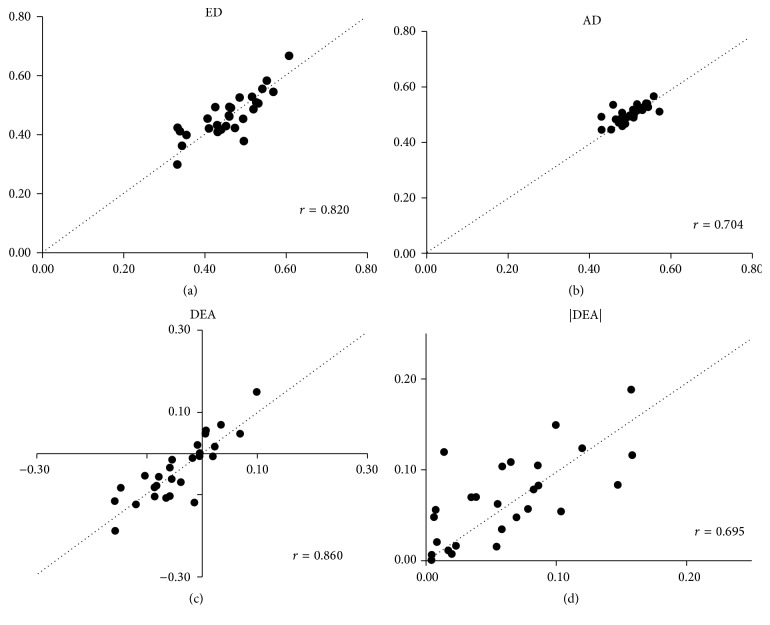
Distribution of each value in PD. The vertical axis shows severer side (SS) [cm/cm], and the horizontal axis shows milder side (MS) [cm/cm]. Dotted line shows *x* = *y* line. The value distributed on the top left (above *x* = *y* line) shows SS is larger than MS. In contrast, the value distributed on the bottom right (below *x* = *y* line) shows MS is larger than SS.

In the control group, the correlation coefficients for each value obtained from the dominant and nondominant sides were *r* = 0.907 (*p* < 0.001) for ED, *r* = 0.620 (*p* < 0.001) for AD, *r* = 0.930 (*p* < 0.001) for DEA, and *r* = 0.792 (*p* < 0.001) for the absolute values of DEA. All variables showed significantly positive correlation. The PD group included 9 patients with SS at the right side and MS at the left side and 18 patients with SS at the left side and MS at the right side. In the PD group, the correlation coefficients for each value obtained at the MS and SS sides were *r* = 0.820 (*p* < 0.001) for ED, *r* = 0.704 (*p* < 0.001) for AD, *r* = 0.860 (*p* < 0.001) for DEA, and *r* = 0.695 (*p* < 0.001) for the absolute values of DEA. All variables showed significantly positive correlation (Figure 2). According to directions, no significant differences in ED, AD, and DEA or in the absolute values of DEA were observed either between the dominant and nondominant sides in the control group or between the MS and SS sides in the PD group. Moreover, the values obtained from measurement in the front direction did not significantly differ from those obtained from measurement in the right and left directions (Table 1).

### 3.4. Relationship between the AD and the ED in the Two Groups

**Figure 3:**
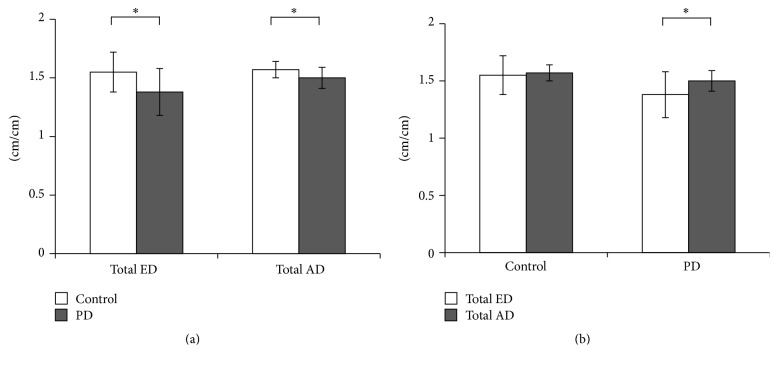
(a) Means and standard deviations demonstrated by each group during total ED and total AD. The PD group showed small value compared with control in both total ED and total AD. (b) Comparison between total ED and total AD within each group is shown. Only the PD group showed a significant difference (^*∗*^ *p* < 0.012).

The mean total AD (1.57 ± 0.07 cm/cm in the control group and 1.50 ± 0.09 cm/cm in the PD group) showed a significant difference between the two groups (total AD: *t*(53) = 3.246, *p* = 0.002) (Figure 3(a)). The mean total ED (1.55 ± 0.17 cm/cm in the control group and 1.39 ± 0.20 cm/cm in the PD group) showed a significant difference between the two groups (total ED: *t*(53) = 3.337, *p* = 0.002) (Figure 3(a)). No significant differences between the total AD and the total ED were observed in control group (*t*(27) = 0.722, *p* = 0.476) (Figure 3(b)). A significant difference between the total AD and the total ED was observed in PD group (*t*(26) = 3.165, *p* = 0.004) (Figure 3(b)).

### 3.5. Association with Clinical Symptoms of PD

**Figure 4:**
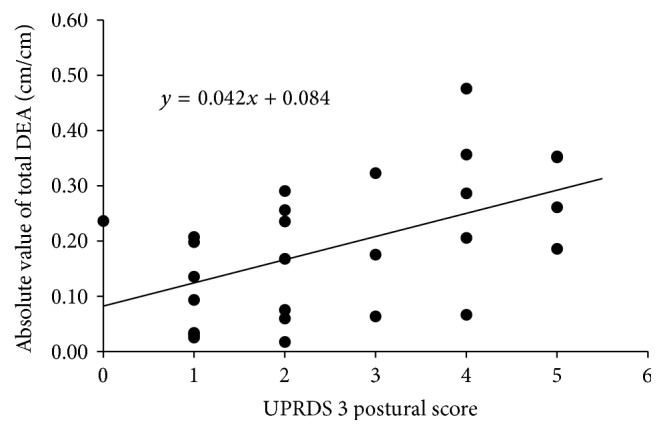
This figure indicates a regression line obtained by multiple regression analysis for the absolute value of total DEA. Postural score was shown to be the only significant factor (*p* = 0.006).

Although clinical features associated with total DEA were investigated, all items were not included as significant factors for the total DEA in the PD group. On the other hand, the result of the stepwise multiple regression analysis for the absolute value of total DEA, posture, was shown to be the only significant factor (*R* ^2^ = 0.268, *β* = 0.518, *p* = 0.006). A relationship between the posture and the absolute value of total DEA is shown in Figure 4. MMSE, disease duration, Hoehn-Yahr scale, history of falls, UPDRS3 total score, tremor, rigidity, diadochokinesis, walking, and akinesia were not included as significant factors for the absolute value of total DEA.

## 4. Discussion

### 4.1. What Does DEA Indicate?

To evaluate distance cognition in a more actual milieu, we measured ED and AD in three directions. ED and DEA are values determined through cognitive processing and may be affected by hand dominance and the order of measurement. Thus, we first measured ED and AD in not only the front direction but also the right and left directions. Then, we examined whether there was any variance in the measurements with the dominant hand, nondominant hand, severity of symptoms, and order of measurement. All values obtained in each reaching direction showed correlations between the right and left sides (Figure 2), and no difference was observed in the mean values of each variable (Table 1). Thus, ED and DEA are assumed to be free from the effects of reaching direction and hand dominance. In the PD patients, these values were not affected by the differences in the severity of motor dysfunction between the right and left sides. This result may support the report that spatial distance was not affected by the difference between right and left symptoms [22, 25]. Furthermore, as Table 1 shows that the values obtained in the front direction do not differ from those obtained in the right or left directions (the dominant and nondominant sides in the controls and the symptomatically milder and severer sides in the PD patients [right SS : left SS = 9 : 18]), the values are also assumed to be free from the effects of the order of measurement. ED, which is considered to be determined on the basis of sensory information and past experience, is affected by environmental factors, such as position of a target object, and physical factors, such as postural stability, body constitution, and flexibility [4–6]. While information on the spatial and positional relationship based on interactions between the environment and the motor system of the body is stored in the brain, this spatial and positional information is considered to be necessary for reaching movement [3]. ED indicates perception of such spatial and positional relationships, and it is assumed that the accuracy of perception can be determined by DEA and absolute value of DEA. Thus, as described in the following section, the characteristics of the PD patients were assessed with regard to cognition of spatial distance.

### 4.2. Stability Limits and Cognition of the Limits in the PD Patients

According to comparisons of total AD, the reaching distance is shorter in the PD patients than in the controls, and the range where the point vertically projected from the center of gravity can be kept within the base of support (stability limit) is shown to be small in the PD patients. This result is consistent with the results reported in many studies [10, 28–30]. In PD, it is considered that the stability limit becomes smaller because of mobility being reduced by rigidity, impaired postural reaction, impaired motion perception, and so forth [28–30].

Moreover, total ED was also smaller in the PD patients than in the controls, showing that the range of stability limit recognized by the PD patients was small. As described above, it is assumed that ED is affected by environmental and physical factors. However, the present study focused on the differences in ED due to physical factors while the environmental factors, such as direction of reaching movement and height of a target object, were kept constant. In the present study, the physical factors include the cognitive process in which people perceive the distance to a target object, compare this distance to their physical status, and determine whether they can reach the target object. ED obtained in the present study might have been affected by the physical characteristics of the participants under these conditions. It can be assumed that the range of stability limit recognized by PD patients on the basis of their physical characteristics is small.

### 4.3. Do PD Patients Underestimate Spatial Distance?

In the PD group, if both AD and ED have decreased equally, it can be assumed that there is no error in cognition of distance. In order to evaluate error in cognition, AD and ED were compared within each group. The results showed that ED tended to be negative compared to AD in the PD group, in other words, showing that the PD group tended to underestimate reaching distance. While people presumably estimate reaching distance on the basis of information from the visual, auditory, and somatic senses and their past experiences in the cognitive process before executing reaching movement [2, 3], PD patients underestimate reaching distance in this process. In PD patients, because it has been reported that their perception of the range of motion of the upper limbs [31] and joint motion angle [32] is lower than the actual values, underestimation of motion perception may affect estimation of spatial distance. Moreover, other studies [33, 34] reported that the decreased range of motion in PD patients is attributable to the lack of perception of their limited motion. Underestimation by the PD group as observed in the present study suggests that PD patients cannot perceive that the distance recognized by them is smaller than actual distance.

Regarding the error in cognition of reaching distance that has been described above, Kamata et al. [9] reported that PD patients tend to overestimate reaching distance. Their result contradicts that of the present study. The reasons for this may be the following 2 factors. One factor is the differences in patient groups. Our results show that although some PD patients overall underestimated the reaching distance, some patients overestimated it. Distribution of MS-DEA and SS-DEA shows the same tendency (Figure 2; DEA, underestimation: the third quadrant, overestimation: the first quadrant). Similarly, the results obtained by Kamata et al. also included overestimation and underestimation values. Depending on the distribution of patients participating in a study, results may show either tendency. The other possible factor is the differences in methods to measure ED. A major difference involves whether the target object is moved or whether participants move during measurement of ED. Ehgoetz Martens et al. [24] investigated the differences in perception of spatial distance by PD patients between the static condition in which they perceived distance from only visual information without moving and the dynamic condition in which they moved to perceive distance. On the basis of these results, Ehgoetz Martens et al. reported that distance is more likely to be underestimated under the dynamic condition than under the static condition. Similarly, Kabasakalian et al. [22] reported hypometric estimates of distance under the dynamic condition. Although the static condition is used in many studies [4–10] including that of Kamata et al., the dynamic condition was used in the present study. This difference in the conditions may cause an underestimation of the reaching distance. Because reaching movements performed in daily life are based on the dynamic condition, the present study may provide important findings with regard to risk factors for falls.

### 4.4. Error in Cognition of Reaching Distance in PD Patients and Causes of the Error

We investigated the factors affecting impaired cognition of distance in PD patients. Because UPDRS3 total scores or Hoehn-Yahr scale were not included as significant factors for both DEA and absolute values of DEA, impaired cognition of reaching distance is not assumed to be necessarily associated with progression of the disease, because all items were not included as significant factors for DEA, which means the factors that affect underestimation of spatial distance were not identified. The tendency of underestimation seems to be affected by the conditions discussed in the previous section rather than clinical symptoms of PD. Meanwhile, it was suggested that postural factors (i.e., posture and postural stability) greatly contributed to the large absolute values of DEA. In PD patients, it has been reported that their perception of body axis is impaired [35, 36]. Moreover the other study reported that impaired spatial cognition correlates with postural factors of UPDRS [37]. Changes in the body axis due to the abnormally flexed posture of PD patients may affect errors in cognition of distance. Moreover, one of the reported factors affecting error in cognition of reaching distance is postural stability. It has been reported that, without the certainty of a stable posture, the error increases [4, 5, 38]. Thus, abnormal posture and impaired postural stability in PD patients appear to be associated with perception of spatial information on the position of their bodies and affect their decisions regarding distance.

In this study, measurements were taken only in on-stage. Levodopa may have affected the results of this study because levodopa improves motor functions of PD [35, 39, 40]. Moreover, some researchers reported that levodopa affects perception of movement and space [24, 41–43]. The results of this study showed that spatial cognition of PD patients was impaired even under the effects of levodopa.

### 4.5. Limitations

While the participants in the present study appear to be adequately distributed between mild and moderate severity of PD, the inclusion criteria have been set to ensure the reliability and validity of measurement methods. Although the clinical symptoms of the PD patients widely varied, it was difficult to examine all symptoms and their severity in the present study. Thus, the abnormal cognition of reaching distance observed in the present study may not be applicable to all PD patients.

## Figures and Tables

**(a):** 

		Front	Dominant	Nondominant	
	ED cm/cm	0.51 ± 0.05	0.52 ± 0.06	0.52 ± 0.06	n.s.
Healthy elderly	AD cm/cm	0.53 ± 0.03	0.52 ± 0.03	0.52 ± 0.02	n.s.
*n* = 28	DEA cm/cm	−0.02 ± 0.04	0.00 ± 0.05	−0.01 ± 0.06	n.s.
	|DEA| cm/cm	0.04 ± 0.03	0.04 ± 0.03	0.05 ± 0.03	n.s.

**(b):** 

		Front	Milder side: MS	Severer side: SS	

	ED cm/cm	0.47 ± 0.08	0.47 ± 0.08	0.47 ± 0.09	n.s.
PD	AD cm/cm	0.50 ± 0.03	0.50 ± 0.03	0.50 ± 0.03	n.s.
*n* = 27	DEA cm/cm	−0.04 ± 0.06	−0.04 ± 0.07	−0.04 ± 0.07	n.s.
	|DEA| cm/cm	0.07 ± 0.05	0.06 ± 0.05	0.07 ± 0.05	n.s.

Mean ± SD [cm/cm].

The one-way ANOVA was performed with each item.

ED: estimated distance, AD: actual distance, DEA: difference between ED and AD, |DEA|: absolute value of DEA, and n.s.: not significant.

## References

[1] Duncan P. W., Weiner D. K., Chandler J., Studenski S. (1990). Functional reach: a new clinical measure of balance. *Journals of Gerontology*.

[2] Andersen R. A., Buneo C. A. (2002). Intentional maps in posterior parietal cortex. *Annual Review of Neuroscience*.

[3] Shumway-Cook A., Woollacott M. (2007). Normal reach, grasp, and manipulation. *Motor Control*.

[4] Carello C., Grosofsky A., Reichel F. D., Solomon H. Y., Turvey M. T. (1989). Visually perceiving what is reachable. *Ecological Psychology*.

[5] Fischer M. H. (2000). Estimating reachability: whole body engagement or postural stability?. *Human Movement Science*.

[6] Robinovitch S. N. (1998). Perception of postural limits during reaching. *Journal of Motor Behavior*.

[7] Robinovitch S. N., Cronin T. (1999). Perception of postural limits in elderly nursing home and day care participants. *Journal of Gerontology, Biological Sciences A*.

[8] Gabbard C., Ammar D., Lee S. (2006). Perceived reachability in single- and multiple-degree-of-freedom workspaces. *Journal of Motor Behavior*.

[9] Kamata N., Matsuo Y., Yoneda T., Shinohara H., Inoue S., Abe K. (2007). Overestimation of stability limits leads to a high frequency of falls in patients with Parkinson's disease. *Clinical Rehabilitation*.

[10] Ryckewaert G., Luyat M., Rambour M. (2015). Self-perceived and actual ability in the functional reach test in patients with Parkinson's disease. *Neuroscience Letters*.

[11] Castiello U., Stelmach G. E., Lieberman A. N. (1993). Temporal dissociation of the prehension pattern in Parkinson's disease. *Neuropsychologia*.

[12] Flash T., Inzelberg R., Schechtman E., Korczyn A. D. (1992). Kinematic analysis of upper limb trajectories in Parkinson's disease. *Experimental Neurology*.

[13] Poizner H., Feldman A. G., Levin M. F. (2000). The timing of arm-trunk coordination is deficient and vision-dependent in Parkinson's patients during reaching movements. *Experimental Brain Research*.

[14] Behrman A. L., Light K. E., Flynn S. M., Thigpen M. T. (2002). Is the functional reach test useful for identifying falls risk among individuals with Parkinson's disease?. *Archives of Physical Medicine and Rehabilitation*.

[15] Smithson F., Morris M. E., Iansek R. (1998). Performance on clinical tests of balance in Parkinson's disease. *Physical Therapy*.

[16] Armstrong R. A. (2011). Visual symptoms in Parkinson's Disease. *Parkinson's Disease*.

[17] Matsumoto H., Terao Y., Furubayashi T. (2011). Small saccades restrict visual scanning area in Parkinson's disease. *Movement Disorders*.

[18] Goyal V., Behari M., Srivastava A., Sood S. K., Shukla G., Sharma R. (2014). Saccadic eye movements in Parkinson's disease. *Indian Journal of Ophthalmology*.

[19] Baziyan B. K., Chigaleichik L. A., Teslenko E. L., Lachinova D. R. (2007). Analysis of trajectories of eye, head, and hand movements for early diagnosis of Parkinson's disease. *Bulletin of Experimental Biology and Medicine*.

[20] Waterston J. A., Barnes G. R., Grealy M. A., Collins S. (1996). Abnormalities of smooth eye and head movement control in Parkinson's disease. *Annals of Neurology*.

[21] Srulijes K., Mack D. J., Klenk J. (2015). Association between vestibulo-ocular reflex suppression, balance, gait, and fall risk in ageing and neurodegenerative disease: protocol of a one-year prospective follow-up study. *BMC Neurology*.

[22] Kabasakalian A., Kesayan T., Williamson J. B. (2013). Hypometric allocentric and egocentric distance estimates in parkinson disease. *Cognitive and Behavioral Neurology*.

[23] Skidmore F. M., Drago V., Pav B., Foster P. S., Mackman C., Heilman K. M. (2009). Conceptual hypometria? An evaluation of conceptual mapping of space in Parkinson's disease. *Neurocase*.

[24] Ehgoetz Martens K. A., Ellard C. G., Almeida Q. J. (2013). Dopaminergic contributions to distance estimation in Parkinson's disease: a sensory-perceptual deficit?. *Neuropsychologia*.

[25] Verreyt N., Nys G. M. S., Santens P., Vingerhoets G. (2011). Cognitive differences between patients with left-sided and right-sided Parkinson's disease. A review. *Neuropsychology Review*.

[26] Karádi K., Lucza T., Ács P. (2015). Visuospatial impairment in Parkinson’s disease: the role of laterality. *Laterality*.

[27] Newton R. A. (2001). Validity of the multi-directional reach test: a practical measure for limits of stability in older adults. *Journals of Gerontology—Series: A Biological Sciences and Medical Sciences*.

[28] Horak F. B., Nutt J. G., Nashner L. M. (1992). Postural inflexibility in parkinsonian subjects. *Journal of the Neurological Sciences*.

[29] Mancini M., Rocchi L., Horak F. B., Chiari L. (2008). Effects of Parkinson's disease and levodopa on functional limits of stability. *Clinical Biomechanics*.

[30] Horak F. B., Dimitrova D., Nutt J. G. (2005). Direction-specific postural instability in subjects with Parkinson's disease. *Experimental Neurology*.

[31] Klockgether T., Borutta M., Rapp H., Spieker S., Dichgans J. (1995). A defect of kinesthesia in Parkinson's disease. *Movement Disorders*.

[32] Zia S., Cody F., O'Boyle D. (2000). Joint position sense is impaired by Parkinson's disease. *Annals of Neurology*.

[33] Farley B. G., Koshland G. F. (2005). Training BIG to move faster: the application of the speed-amplitude relation as a rehabilitation strategy for people with Parkinson's disease. *Experimental Brain Research*.

[34] Fox C., Ebersbach G., Ramig L., Sapir S. (2012). LSVT LOUD and LSVT BIG: Behavioral treatment programs for speech and body movement in Parkinson disease. *Parkinson's Disease*.

[35] Wright W. G., Gurfinkel V. S., King L. A., Nutt J. G., Cordo P. J., Horak F. B. (2010). Axial kinesthesia is impaired in Parkinson's disease: effects of levodopa. *Experimental Neurology*.

[36] Konczak J., Corcos D. M., Horak F. (2009). Proprioception and motor control in Parkinson's disease. *Journal of Motor Behavior*.

[37] Murakami H., Owan Y., Mori Y. (2013). Correlation between motor and cognitive functions in the progressive course of Parkinson's disease. *Neurology and Clinical Neuroscience*.

[38] Gabbard C., Cordova A., Lee S. (2007). Examining the effects of postural constraints on estimating reach. *Journal of Motor Behavior*.

[39] Parkinson Study Group (2000). Pramipexole vs levodopa as initial treatment for Parkinson disease. *The Journal of the American Medical Association*.

[40] Parkinson Study Group (2004). Pramipexole vs Levodopa as initial treatment for Parkinson disease: a 4-year randomized controlled trial. *Archives of Neurology*.

[41] Barnett-Cowan M., Dyde R. T., Fox S. H., Moro E., Hutchison W. D., Harris L. R. (2010). Multisensory determinants of orientation perception in Parkinson's disease. *Neuroscience*.

[42] Konczak J., Krawczewski K., Tuite P., Maschke M. (2007). The perception of passive motion in Parkinson's disease. *Journal of Neurology*.

[43] Maschke M., Gomez C. M., Tuite P. J., Konczak J. (2003). Dysfunction of the basal ganglia, but not the cerebellum, impairs kinaesthesia. *Brain*.

